# Defense responses of arbuscular mycorrhizal fungus-colonized poplar seedlings against gypsy moth larvae: a multiomics study

**DOI:** 10.1038/s41438-021-00671-3

**Published:** 2021-12-01

**Authors:** Dun Jiang, Mingtao Tan, Shuai Wu, Lin Zheng, Qing Wang, Guirong Wang, Shanchun Yan

**Affiliations:** 1 School of Forestry, Northeast Forestry University, 150040 Harbin, P. R. China; 2 Key Laboratory of Sustainable Forest Ecosystem Management-Ministry of Education, Northeast Forestry University, 150040 Harbin, P. R. China; 3 State Key Laboratory for Biology of Plant Diseases and Insect Pests, Institute of Plant Protection, Chinese Academy of Agricultural Sciences, Beijing, P. R. China

**Keywords:** Plant physiology, Herbivory

## Abstract

Arbuscular mycorrhizal (AM) fungi may help protect plants against herbivores; however, their use for the pest control of woody plants requires further study. Here, we investigated the effect of *Glomus mosseae* colonization on the interactions between gypsy moth larvae and *Populus alba* *×* *P. berolinensis* seedlings and deciphered the regulatory mechanisms underlying the mycorrhizal-induced resistance in the leaves of mycorrhizal poplar using RNA-seq and nontargeted metabolomics. The resistance assay showed that AM fungus inoculation protected poplar seedlings against gypsy moth larvae, as evidenced by the decreased larval growth and reduced larval survival. A transcriptome analysis revealed that differentially expressed genes (DEGs) were involved in jasmonic acid biosynthesis (lipoxygenase, hydroperoxide dehydratase, and allene oxide cyclase) and signal transduction (jasmonate-ZIM domain and transcription factor MYC2) and identified the genes that were upregulated in mycorrhizal seedlings. Except for chalcone synthase and anthocyanidin synthase, which were downregulated in mycorrhizal seedlings, all DEGs related to flavonoid biosynthesis were upregulated, including 4-coumarate-CoA ligase, chalcone isomerase, flavanone 3-hydroxylase, flavonol synthase, and leucoanthocyanidin reductase. The metabolome analysis showed that several metabolites with insecticidal properties, including coumarin, stachydrine, artocarpin, norizalpinin, abietic acid, 6-formylumbelliferone, and vanillic acid, were significantly accumulated in the mycorrhizal seedlings. These findings suggest the potential of mycorrhiza-induced resistance for use in pest management of woody plants and demonstrate that the priming of JA-dependent responses in poplar seedlings contributes to mycorrhiza-induced resistance to insect pests.

## Introduction

Arbuscular mycorrhizal (AM) fungi (AMF) that belong to the subphylum Glomeromycotina occur in soil and are widely distributed in agroforestry ecosystems. Fossil records and molecular data show that AMF coevolved with terrestrial plants and their symbiotic relationship was established 450 million years ago^1^. Arbuscular mycorrhizae represent the most common form of symbiosis between AMF and host plants, with associations observed with ~80% of terrestrial vascular plants^2^. AMF hyphae typically penetrate the root epidermis to colonize cortical cells and then form arbuscules composed of highly branched hyphae ensheathed in a plant-derived membrane termed the periarbuscular membrane^3,4^. Through AM symbiosis, AMF can transfer absorbed water and mineral nutrients (e.g., phosphate and nitrogen) to the host plant; in exchange, the host plant transports carbohydrates synthesized by photosynthesis to AMF in the form of hexose, which is used as the carbon source of fungi^5^. This mutualistic association promotes material exchange and information transmission between AMF and host plants, thereby achieving an optimal symbiotic state by effectively controlling the nutrient balance between the host and the fungi^3,5^. Intriguingly, AM symbiosis triggers a systemic effect on underground and epigeous portions of plants beyond nutritional benefits, including improving photosynthetic processes, promoting changes in primary and secondary metabolism, enhancing reactive oxygen scavenging ability, and maintaining water uptake, osmotic potential and cell membrane integrity^6,7^. These alterations imply that root colonization by AMF not only affects plant growth but can also regulate plant responses to biotic (e.g., bacterial pathogens and nematodes) or abiotic stress (e.g., climatic changes, water deficit, and heavy metal pollution)^8^.

During their lengthy interaction with phytophagous insects, plants have evolved defense mechanisms to reduce herbivore damage. Plant resistance to insects can be divided into constitutive resistance and inducible resistance. Inducible resistance is characterized by its broad spectrum and rapid effects; moreover, it can enhance the defense ability of plants against insect attacks when stimulated by specific factors^9,10^. Currently, inducible resistance is a research hotspot in phytochemical ecology. Many studies have demonstrated that inducible resistance in plants can be triggered by biotic stimuli, such as nonpathogenic microbial infection and pest feeding, and abiotic stimuli, such as mechanical damage and hormone treatment^11,12^. Studies on mycorrhizae have shown that AMF inoculation, as a biotic stimulus, can also activate inducible resistance and protect plants against herbivorous insects and bacteria^13–15^. For example, Schoenherr et al.^16^ reported that root colonization by the AM fungus *Rhizophagus irregularis* improved potato resistance to the cabbage looper (*Trichoplusia ni*), as evidenced by decreases in the weight gain of larvae that fed on mycorrhizal plants compared with nonmycorrhizal plants. Further research has led to the proposal of the “mycorrhizal-induced resistance” hypothesis to explain the boost in basal defenses in mycorrhizal plants^17^. Several studies have revealed that mycorrhizal-induced resistance appears to be a cumulative effect of mycorrhizal colonization on the plant that involves alteration of plant morphology, activation of the jasmonic acid (JA) signaling pathway, and biosynthesis of defense metabolites (e.g., flavonoids)^18,19^. With regard to its cost-effective and sustainable solution for plant pest control, mycorrhizal symbiosis has been regarded as an alternative to chemical pesticides and fertilizers in sustainable agricultural practices. However, positive or neutral effects of mycorrhizal symbiosis on the performance of phytophagous insects, especially specialist-chewing insects, have also been observed in several mycorrhizal plants^20–22^. Thus, it appears that the outcome of the mycorrhizal plant-herbivore interaction may vary with the AMF, host plant, and insect species involved as well as environmental factors.

Since root colonization by AMF can modify the pairwise interactions between plants and phytophagous insects, it would be useful to expand the application potential of mycorrhizal-induced resistance in plant protection and to better understand the mechanism by which AMF affect plant resistance to insects. There are many mycorrhiza-related studies that have focused on plants, such as rice, tomato, potato, wheat, and sorghum sudangrass^15,16,23,24^. However, these studies mainly focused on cultivated crops, and less information has been obtained on the tripartite interaction involving an AM fungus, a woody plant and a phytophagous insect. Furthermore, little is known about the systemic transcriptome and metabolome phenotypes in mycorrhizal plants that affect plant interactions with aboveground herbivores.

*Populus alba* × *P. berolinensis* is widely distributed in Northeast China, and it is an excellent tree species for fast-growing and high-yield plantations and a preferred species for shelter-belt forests, water conservation forests, and urban ornamental trees. The Asian gypsy moth (*Lymantria dispar*) is an economically important insect pest in the Asia-Pacific region that can also be used as an excellent biological indicator for assessing plant resistance to insects due to its well-known developmental stages and short life cycle^25^. In the present study, we investigated whether inoculation with a commercial inoculum containing a single *Glomus mossae* strain affects poplar (*P. alba* × *P. berolinensis*) seedling growth and its resistance to gypsy moth larvae. Furthermore, we integrated transcriptome and metabolomic analyses of mycorrhizal plant leaf tissues to determine the regulatory mechanism underlying the effect of AM symbiosis on poplar resistance to gypsy moth larvae at the translational and metabolic levels. Using this factorial design, we examined the following possibilities: (1) colonization of roots by AM fungus can promote generalist-chewing insect resistance in poplar seedlings; (2) priming of JA-dependent responses through the JA biosynthesis and signal transduction pathways as well as the flavonoid biosynthesis pathway may promote mycorrhizal-induced insect resistance in poplar seedlings. Relevant findings in this study could improve our understanding of the tripartite interaction among woody plants, generalist-chewing insects, and AM fungi and enhance the application potential of “mycorrhiza-induced resistance” for pest management of woody plants.

## Results

### AM fungal root colonization and growth evaluation of poplar seedlings

A colonization analysis revealed that the commercial mycorrhizal inoculum *G. mosseae* succeeded in establishing a mutualistic association with *P. alba* × *P. berolinensis* seedlings, with a 72.46% mycorrhizal infection rate in the GM group (Fig. S1A and B). Compared to the nonmycorrhizal plants, AM symbiosis in the poplar seedlings significantly improved the root length and root fresh/dry weights but had no significant effects on the plant height or the fresh/dry weights of the aboveground parts (Table S1).

### Resistance of mycorrhizal poplar plants to gypsy moth larvae

**Fig. 1:**
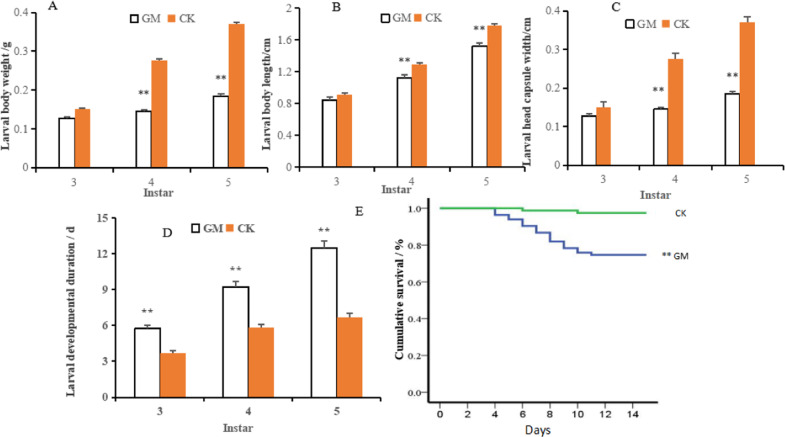
Growth and survival rate of gypsy moth larvae fed on the leaves of mycorrhizal or nonmycorrhizal poplar plants. Larval body weight (**A**). Larval body length (**B**). Larval head capsule width (**C**). Larval developmental duration (**D**). Larval survival curve (**E**). Values in the growth parameters are given as the means + standard deviations (*n* = 24). Asterisks above the bars indicate significant differences among groups (for (**A**–**D**), *t* test, *n* = 24; for **E**, log-rank test, *n* = 80): **P < 0.01. GM *Glomus mossae*-treated group, CK untreated group.

To study the resistance of woody plants that were colonized with AM fungus to insects, we analyzed the growth and survival of gypsy moth larvae that fed on the leaves of *P. alba* × *P. berolinensis* seedlings in which the roots were colonized with *G. mosseae* or control leaves from plants without mycorrhizal colonization. Compared to the control, the AM fungus treatment significantly decreased the body weights/lengths and head capsule widths of the 4th and 5th instar gypsy moth larvae and prolonged the development of the 3rd–5th instar larvae (Fig. 1A–D). The larval survival rate in the GM group was significantly lower than that in the CK group, as shown in the survival curve (Fig. 1E and Table S2).

### Overview of the RNA-seq and functional analyses of DEGs

To decipher the resistance response of *P. alba* × *P. berolinensis* seedlings to gypsy moth larvae at the molecular level that were affected by AM symbiosis, genome-wide transcript abundances in the seedling leaves were compared between the GM group and the CK group. A total of 127,960 transcripts and 46,397 genes were obtained from the six leaf samples using the Illumina HiSeq High-throughput sequencing platform (Illumina, USA), with average lengths of 1057 bp and 957 bp (ranging from 201 bp to 16491 bp) and N50 values of 1566 bp and 1597 bp, respectively (Table S3). A quality analysis of the transcriptome sequencing showed that mycorrhizal and nonmycorrhizal plants had average GC (%) contents of 45.72% and 45.76% clean reads, Q20 contents of 98.26% and 98.31%, and Q30 contents of 94.46% and 94.51%, respectively (Table S3). To acquire the overall genetic information, all unigenes were BLASTX searched against six databases, including the KEGG (Kyoto Encyclopedia of Genes and Genomes), eggNOG (Evolutionary Genealogy of Genes: Non-supervised Orthologous Groups), Swiss-Prot (Swiss-Prot protein sequence database), Pfam (Protein family), Nr (Nonredundant protein sequences) and GO (Gene Ontology) databases. The results showed that there were 46397 annotated genes, of which 32.26% were annotated by all six databases and 42.40% matched to at least one public database (Fig. S2). Statistical assessment of differentially expressed unigenes between the GM treatment and CK groups revealed that a total of 3671 genes (FDR < 0.05 and |log_2_FC|> 1; ca. 7.9% of all genes) expressed in the poplar leaves showed differential expression under AM fungus inoculation, including 2427 up- and 1244 down-regulated DEGs (Fig. S3A/B). To further validate the transcriptome data, 12 randomly selected DEGs were analyzed by RT-PCR, and they presented similar expression patterns as those observed in the RNA-seq results (Fig. S4).

To gain insights into the functional categories that differed between the GM and CK groups, GO and KEGG pathway analyses of these DEGs were performed. The GO enrichment analysis classified 3005 DEGs into 2310 terms related to biological processes, cellular components and molecular function, with 368 significantly enriched GO terms (*P* < 0.05). Of these, photosynthesis was the most enriched category annotated for biological processes (Table S4). Thylakoid, chloroplast stroma and chloroplast thylakoid were the major categories annotated under cellular components (Table S4). For the GO terms of the molecular function, the major categories included hydrolase activity, hydrolyzing O-glycosyl compounds and chlorophyll binding (Table S4). The KEGG analysis also assigned 2217 DEGs to 128 pathways (Fig. S5). Of these, 77 were significantly enriched KEGG pathways (*P* < 0.05). Further classification of the KEGG pathways showed that metabolic pathways (e.g., photosynthesis and glyoxylate and dicarboxylate metabolism) accounted for 90.91% of these significantly enriched pathways and contained nine secondary classifications that were composed of carbohydrate metabolism, energy metabolism, lipid metabolism, amino acid metabolism, other amino acid metabolism, glycan biosynthesis and metabolism, metabolism of cofactors and vitamins, terpenoid and polyketide metabolism, and other secondary metabolite biosynthesis.

### Photosynthesis-related DEGs induced by AM symbiosis in leaves

**Fig. 2:**
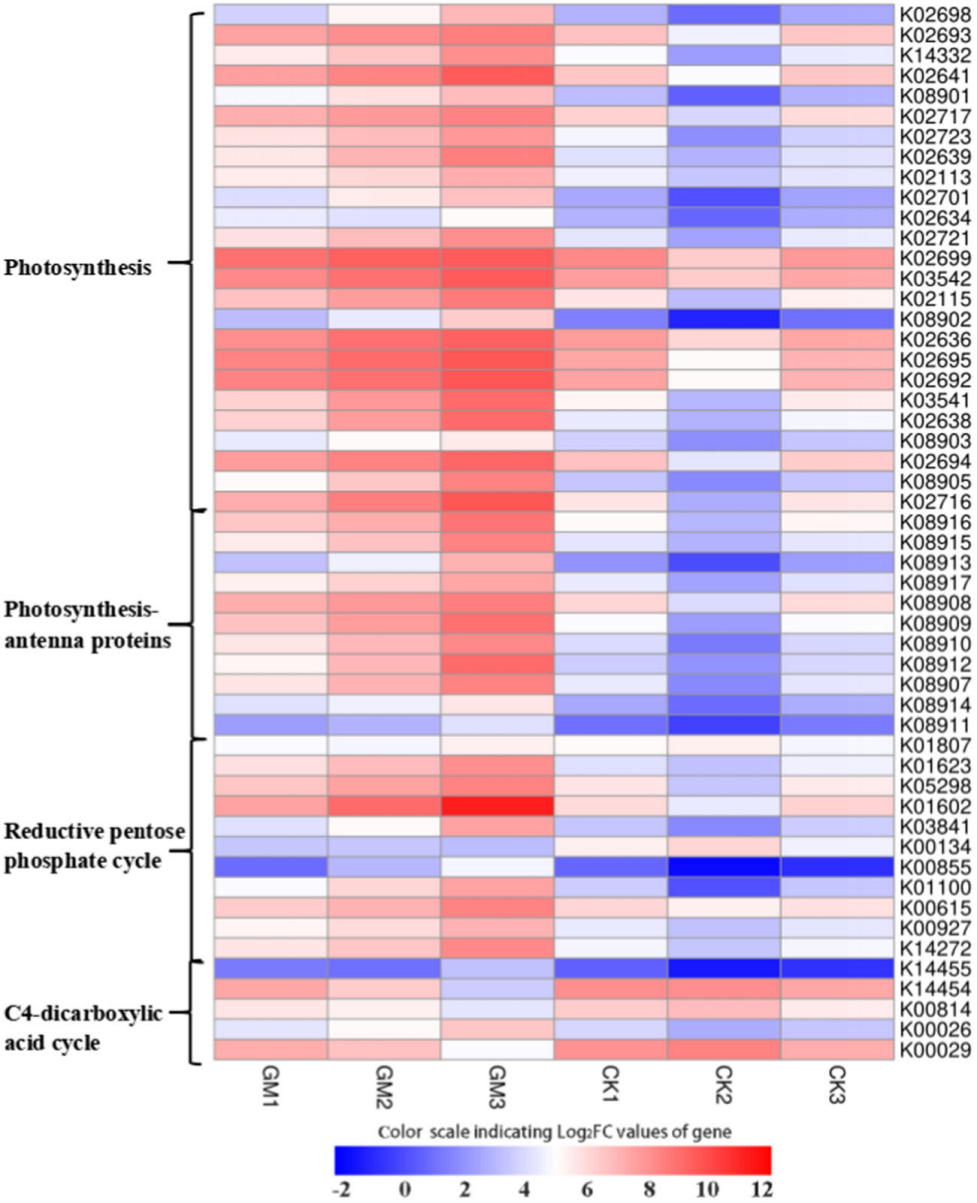
Heatmap of the differentially expressed genes related to photosynthesis in the leaves of mycorrhizal poplar seedlings. GM *Glomus mossae*-treated group, CK untreated group. Heatmap analysis was performed using the OmicStudio tools (https://www.omicstudio.cn/tool).

A total of 130 genes associated with photosynthesis were identified by transcriptome sequencing. Among these, 97 DEGs were considered to be responsive to AM symbiosis in mycorrhizal poplar leaves and were enriched in the following three KEGG pathways: 43 DEGs belonging to photosynthesis, 38 DEGs for carbon fixation in photosynthetic organisms and 16 DEGs belonging to photosynthesis-antenna proteins (Fig. 2). All DEGs related to photosynthesis and photosynthesis-antenna proteins were upregulated, although in the carbon fixation pathway, most DEGs (e.g., NADP-dependent malic enzyme and aspartate aminotransferase) related to the C4-dicarboxylic acid cycle were downregulated while most reductive pentose phosphate cycle-related DEGs were upregulated (Fig. 2).

### Transcription factors involved in the defense of mycorrhizal poplar plants

Given the important regulatory functions of transcription factors (TFs), TF-encoding genes were analyzed by sequence alignment with the Plant Transcription Factor Database (http://plntfdb.bio.uni-potsdam.de/v3.0/). A total of 90 DEGs (44 up- and 46 down-regulated) were identified as TFs, and they were distributed in 14 families and accounted for 5.7% of all the DEGs regulated by AM symbiosis. Of all the differentially expressed TFs, the ERF family showed the largest percentage (~27.8%; 25 DEGs), followed by bHLH (18 DEGs), WRKY (13 DEGs), MYB (11 DEGs), NF-YA (4 DEGs), GATA (3 DEGs), NAC (3 DEGs), Trihelix (3 DEGs), and HSF (3 DEGs). DEGs annotated as members of the NF-YA and GATA families were all upregulated. Most of the AM fungus-responsive TFs belonging to the MYB, BHLH NAC and Trihelix families were upregulated, whereas more than half of the identified TFs from the ERF, WRKY, BZIP, and HSF families were downregulated.

### Identification of DEGs associated with mycorrhizal-induced resistance

**Fig. 3:**
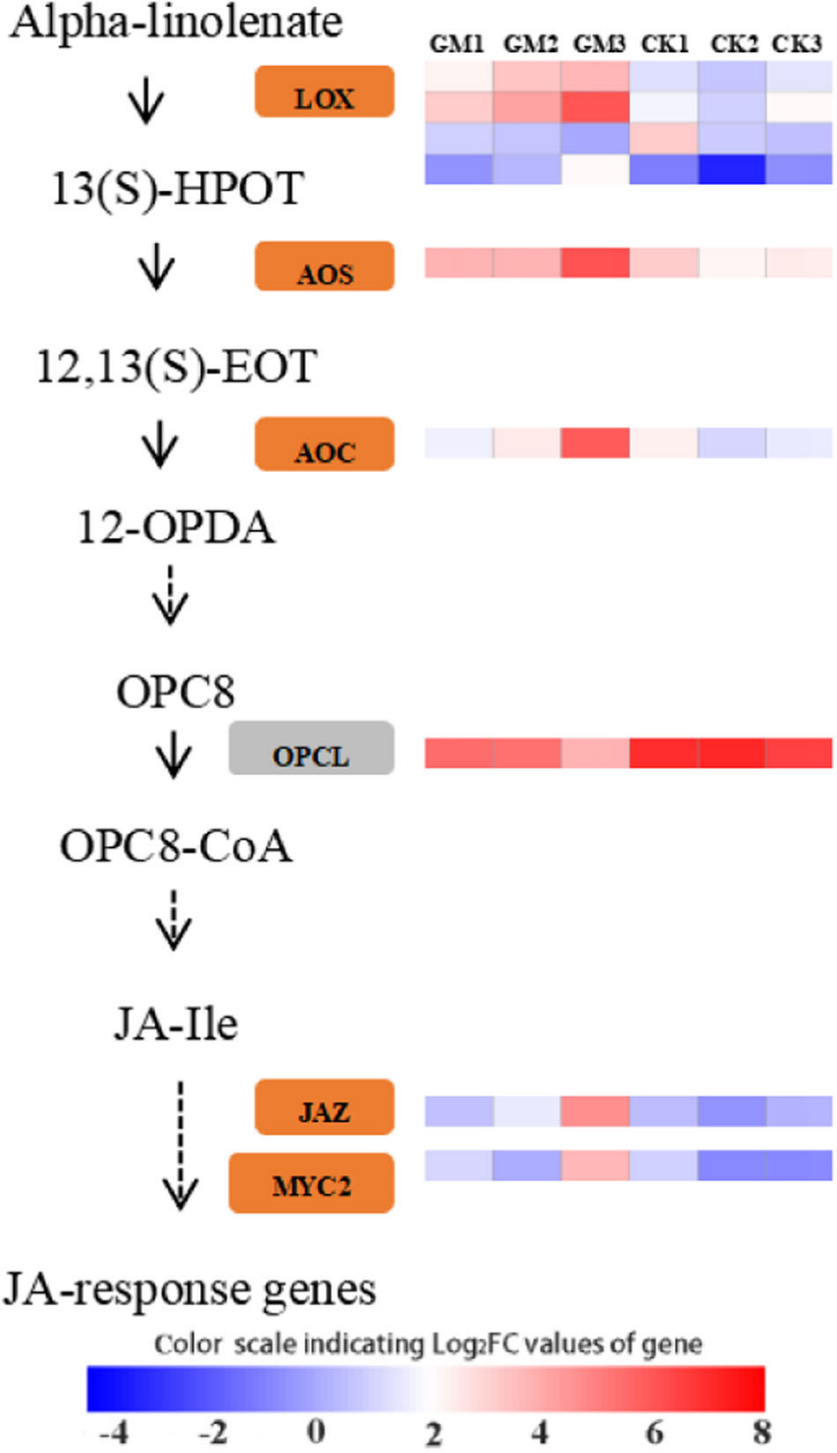
Arbuscular mycorrhizal fungus-induced responses in the jasmonic acid (JA) signaling pathway. The orange and gray boxes indicate upregulated and downregulated genes, respectively. Lipoxygenase (LOX); 13(S)-hydroperoxylinolenic acid (13(S)-HPOT); hydroperoxide dehydratase (AOS); 12,13(S)-epoxylinolenic acid (12,13(S)-EOT); allene oxide cyclase (AOC); 12-oxo-phytodienoic acid (12-OPDA); 3-oxo-2-(2′ (Z)-pentenyl)-cyclopentane-1-octanoic acid (OPC8); OPC8 CoA ligase (OPCL); jasmonoyl-L-isoleucine (JA-Ile); jasmonate-ZIM domain (JAZ); transcription factor MYC2 (MYC2). GM *Glomus mossae*-treated group, CK untreated group. Heatmap analysis was performed using the OmicStudio tools (https://www.omicstudio.cn/tool).

**Fig. 4:**
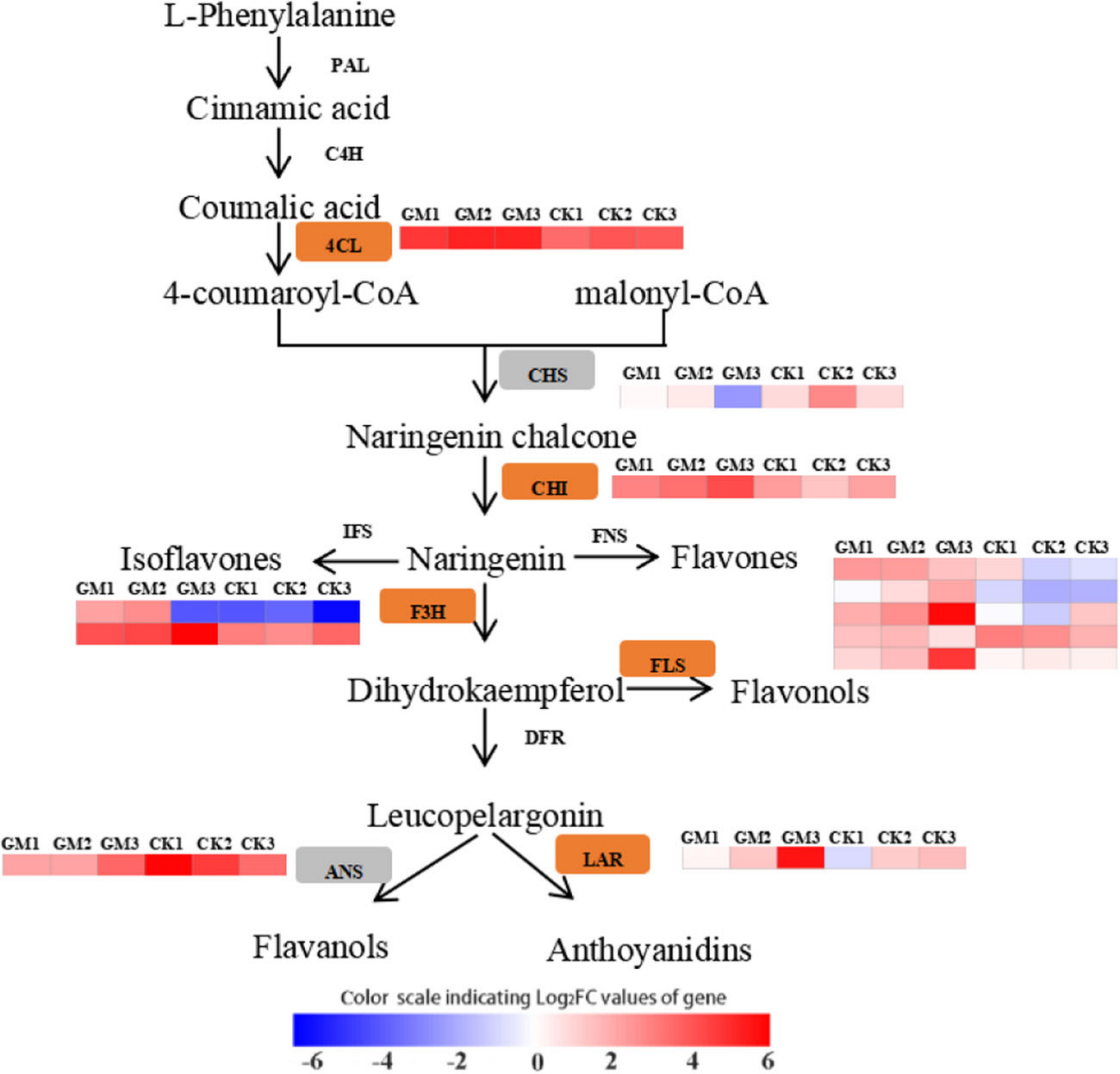
Arbuscular mycorrhizal fungus-induced responses in the flavonoid pathway. The orange and gray boxes indicate that genes are upregulated and downregulated, respectively. Phenylalanine ammonia-lyase (PAL); 4-coumarate-CoA ligase (4CL); cinnamic acid-4-hydroxylase (C4H); chalcone synthase (CHS); chalcone isomerase (CHI); isoflavone synthase (IFS); flavone synthase (FNS); flavanone 3-hydroxylase (F3H); flavonol synthase (FLS); leucoanthocyanidin reductase (LAR); anthocyanidin synthase (ANS). GM *Glomus mossae*-treated group. CK untreated group. Heatmap analysis was performed using the OmicStudio tools (https://www.omicstudio.cn/tool).

To focus on how AM symbiosis influences defense responses related to mycorrhizal-induced resistance, the expression levels of the most likely gene candidates in the jasmonic acid (JA) signaling pathway and flavonoid biosynthesis were depicted. An expression analysis of the CK and GM groups identified a total of six JA signaling pathway-related genes that were responsive to the *G. mossae* treatment, with four genes involved in JA biosynthesis and two genes involved in JA signal transduction (Fig. 3). Among these genes, OPC8 CoA ligase (OPCL) was significantly downregulated while the remaining genes were upregulated in mycorrhizal poplar plants, including lipoxygenase (LOX), hydroperoxide dehydratase (AOS), allene oxide cyclase (AOC), jasmonate-ZIM domain (JAZ), and transcription factor MYC2 (MYC2) (Fig. 3). One gene involved in the formation of cinnamoyl-CoA (an initial metabolite in flavonoid biosynthesis) and six genes involved in flavonoid biosynthesis were identified to be responsive to the AM fungus treatment (Fig. 4). Of these, chalcone synthase (CHS) and anthocyanidin synthase (ANS) genes were significantly downregulated while 4-coumarate-CoA ligase (4CL), chalcone isomerase (CHI), flavanone 3-hydroxylase (F3H), flavonol synthase (FLS), and leucoanthocyanidin reductase (LAR) genes were significantly upregulated upon AM symbiosis (Fig. 4).

### Metabolite analysis of mycorrhizal poplar leaves

To analyze the variation in metabolomic profiles in response to AM symbiosis, a nontargeted metabolomics analysis of poplar leaf samples was conducted via UPLC-QTOF MS in ESI^+ ^mode. A total of 603 metabolites were successfully identified in both nonmycorrhizal and mycorrhizal poplar leaves and could be classified into 58 major classes. Among these metabolites, “flavonoids” (52), “benzene and substituted derivatives” (51), “glycerophospholipids” (40), “organooxygen compounds” (33), “prenol lipids” (27), and “fatty acyls” (24) were the top six accumulated metabolite groups (Fig. S6). A PCA (principal component analysis) of the resulting metabolite data revealed a considerable separation of the GM group from the CK group, with 47.75% and 6.10% variations explained by the first two principal components (PC1 and PC2, respectively) (Fig. S7A). Score plots from the PLS-DA (supervised partial least squared discriminant analysis) model also showed a clear separation between the GM group and the CK group (Fig. S7B). The model parameter R2 and Q2 intercept values analyzed after 200 permutation tests were 0.996 and −0.643, respectively, suggesting that the robustness of the models presented low overfitting and reliability risks (Fig. S7C).

**Fig. 5:**
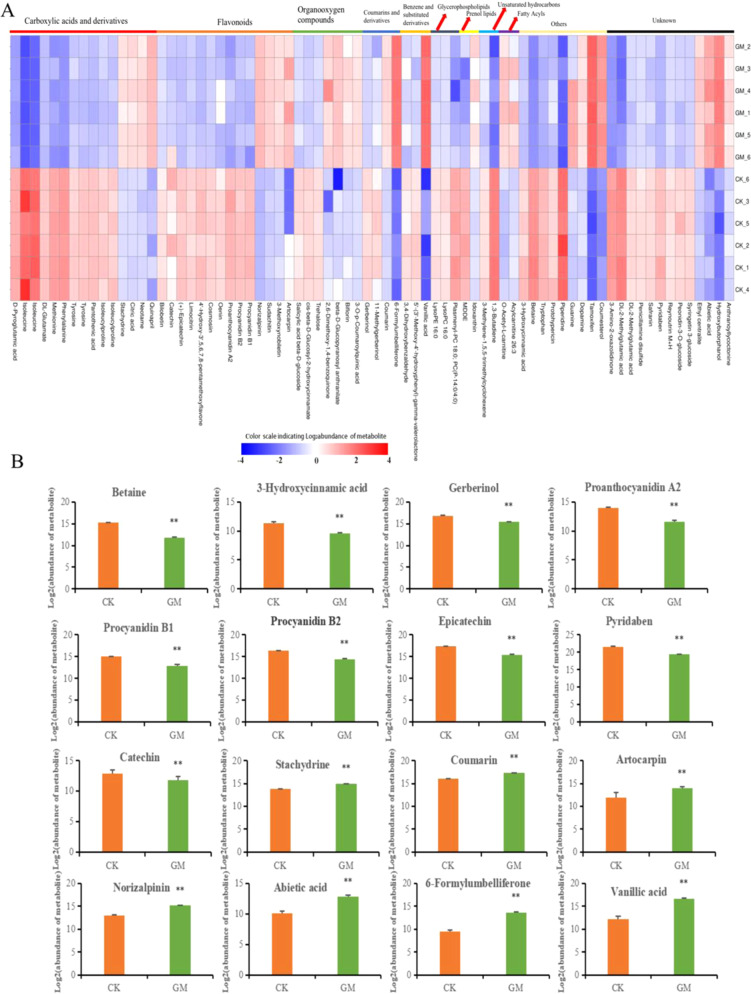
Significantly altered metabolites in the leaves of mycorrhizal poplar seedlings. **A** Heatmap analysis of 74 differentially expressed metabolites between nonmycorrhizal (CK) and mycorrhizal (GM) plants. The figure shows the data processed by the log2 function and mean subtraction in turn. **B** Relative abundance of differentially expressed metabolites with insecticidal properties. Heatmap analysis was performed using the OmicStudio tools (https://www.omicstudio.cn/tool).

By filtering with VIP > 1 and a *P* value < 0.05, discriminant analyses identified up to 74 metabolites that were differentially regulated in the leaves of mycorrhizal poplar seedlings compared with the nonmycorrhizal control poplar seedlings (Fig. 5A). Of these metabolites, the contents of 48 and 26 metabolites were reduced and enriched in the GM group, respectively. The most significantly altered metabolites between the mycorrhizal and nonmycorrhizal plants were carboxylic acids and derivatives and flavonoid compounds. Further in-depth comparative analysis indicated that a majority of metabolites associated with resistance against insects, including flavonoids (e.g., catechin, procyanidin B1, procyanidin B2, proanthocyanidin A2, and epicatechin), carboxylic acid derivatives (e.g., betaine), cinnamic acid derivatives (e.g., 3-hydroxycinnamic acid), and coumarin derivatives (e.g., gerberinol), were decreased in the leaves of the mycorrhizal poplar plants (Fig. 5B). However, the content of several metabolites with insecticidal properties, including coumarin, stachydrine, artocarpin, norizalpinin, abietic acid, 6-formylumbelliferone, and vanillic acid, was significantly increased in the leaves of mycorrhizal poplar plants (Fig. 5B). Through metabolic pathway mapping with the MetaboAnalyst website (https://www.metaboanalyst.ca/), these differentially expressed metabolites were enriched in 42 KEGG pathways (Fig. S8). On the basis of both the lg(*P* value) and pathway impact scores, the most relevant metabolic pathways in response to AM symbiosis were isoquinoline alkaloid biosynthesis, flavone and flavonol biosynthesis, glycerolipid metabolism, phenylalanine metabolism, and glycerophospholipid metabolism (Fig. S8).

## Discussion

AM symbiosis, as a potential broad-spectrum and effective pest control strategy, may represent a valid alternative to some chemical pesticides in contemporary agriculture^26^. Based on the “mycorrhiza-induced resistance” theory, many commercial AMF products have been developed to improve crop resistance or tolerance to insect pests^27^. However, the applicability of this insect resistance mechanism in woody plants is lacking. In the present study, we investigated the outcome of insect–plant–microbe interactions with a focus on *P. alba* × *P. berolinensis* seedlings, gypsy moth larvae, and *G. mossae*. This is the first report on the tripartite interaction among a *Populus* plant, an AM fungus and a generalist-chewing insect. In addition, we tracked the transcriptomic and metabolomic alterations in the leaves of the mycorrhizal and nonmycorrhizal poplar seedlings using RNA-seq and nontargeted metabolomics technologies, which provided us with an opportunity to examine the candidate genes and metabolites related to metabolic pathways underlying the resistance to insects in AM fungus-colonized woody plants.

### Growth evaluation

Augmented plant growth and biomass (e.g., rice and wheat) in response to inoculation with AMF have been repeatedly reported in various laboratory or field experiments, a phenomenon generally identified as a positive mycorrhizal growth response (MGR)^28,29^. Positive MGRs appear to be the consequence of improved uptake and translocation of soil nutrients, generally phosphorus and nitrogen, as the hyphal network can extend the soil area of the mycorrhizal roots to absorb nutrients^24,30^. However, in the present study, under the AM fungus treatment, although the biomass of root tissues was significantly increased, the plant height as well as the fresh and dry weights of the aboveground parts were not significantly different from those of the nonmycorrhizal poplar seedlings. These results clearly suggested a species- or tissue-specific growth response to AM fungi in plants.

The transcriptomic analysis showed that AM symbiosis significantly activated photosynthesis-related pathways in the leaves of the *P. alba* × *P. berolinensis* seedlings. All photosynthesis and photosynthesis antenna protein-related DEGs and most of the reductive pentose phosphate cycle-related DEGs were upregulated. Previous studies demonstrated that during the symbiosis between AMF and plants, AMF competed with the host plants for photosynthates and the carbon consumed by AMF could account for up to 4–20% of photosynthetically assimilated carbon^26,31^. Our results along with the findings of previous studies unambiguously reveal that improved photosynthetic efficiency in plants is a major regulatory mechanism for maintaining a mutualistic association with AMF. In addition, photosynthesis is the most critical factor restricting plant growth, and its improvement usually promotes plant growth. Nevertheless, we observed a neutral MGR for the aboveground biomass (growth) of *P. alba* × *P. berolinensis* seedlings in this study. Therefore, based on the consumption of photosynthates by mycorrhizal colonization, we believe that the neutral MGR may be attributed to the possible negative effects of carbon losses in the host plant caused by AMF inoculation reaching a balance with the benefits in growth acquired from the increased nutrient supply or activated photosynthesis. Notably, growth inhibition in mycorrhizal plants (negative MGR) might even be triggered when the cost of carbon losses to the plant exceeds the benefit in growth acquired from increased nutrient supply. This situation has been previously reported in some cultivated crops^32,33^.

### Resistance response to insects

Systemic protection by mycorrhizal symbiosis can also be found in the aerial parts of AMF-inoculated plants, although the consequence of mycorrhizal fungus-plant-herbivore interactions is variable and may have a positive, negative, or mixed effect^18,34^. Consistent with previous studies in crops, such as alfalfa, rice, wheat, and tomato^15,23,35,36^, our results showed that treating poplar seedlings with AM fungus significantly decreased the survival, body weight, body length, and head capsule width of gypsy moth larvae and significantly prolonged the duration of larval development. This finding indicates that AM fungal colonization increased the resistance of *P. alba* × *P. berolinensis* seedlings to gypsy moth larvae. The present study provides evidence that AM symbiosis can lead to priming in plant tissues for a more rapid defensive response and that mycorrhiza-induced resistance is also applicable to woody plants. It is worth mentioning that the larval survival rate in the GM group remained high while the development duration was greatly increased. Prolonged development of gypsy moth larvae under unfavorable conditions has been reported in several studies, such as under heavy metal stress^37,38^. These results indicate that delaying development might be an effective survival strategy for gypsy moth larvae to obtain sufficient energy for growth under adverse environments.

### Defense response in mycorrhizal poplar seedlings

AMF inoculation can elicit inducible resistance in plants by activating a broad range of defense genes that are expressed only after pathogen or insect attack^16,17^. The key regulators of mycorrhiza-induced resistance are plant hormones, such as jasmonic acid and salicylic acid, which usually accumulate in plants with AMF colonization^18,26^. The JA signaling pathway is an important defense response in many plants^39^; therefore, we systematically elaborated the expression model of genes involved in JA biosynthesis and JA signal transduction in mycorrhizal poplar seedlings. Our results showed that three JA biosynthesis-related genes (LOX2, AOS, and AOC) and two JA signal transduction-related genes (JAZ and MYC2) were upregulated in mycorrhizal seedlings compared to nonmycorrhizal seedlings, and they represent key regulatory genes in the JA signaling pathway. For example, under the continuous catalysis of LOX, AOS, and AOC, alpha-linolenic acid can be transformed into the JA precursor 12-oxo-phytodienoic acid^40^. Therefore, their upregulation at the mRNA level indicates that AM fungus inoculation stimulates the JA signaling pathway in the leaves of *P. alba* × *P. berolinensis* seedlings and demonstrates that activation of the JA signaling pathway is a major component of mycorrhiza-induced resistance. Similar results were reported by Schoenherr et al.^16^, who observed systemic upregulation of the transcript levels of AOS1 and 12-oxo-phytodienoate reductase 3 (belonging to the JA signaling pathway) in potatoes that were colonized with the AM fungus *Rhizophagus irregularis*.

Flavonoids have insecticidal effects and help protect plants against phytophagous insects^41,42^. The biosynthesis of flavonoid compounds is generally divided into the following three stages: *L*-phenylalanine to 4-coumaroyl-CoA, which is catalyzed by PAL, 4CL and cinnamic acid-4-hydroxylase (C4H); 4-coumaroyl-CoA to flavonone, which is catalyzed by CHS and CHI; and flavonone to various types of flavonoids, including isoflavones, which are catalyzed by isoflavone synthase (IFS), flavones, which are catalyzed by flavone synthase (FNS), flavonols, which are catalyzed by FLS, flavanols, which are catalyzed by LAR, and anthoyanidins, which are catalyzed by ANS^43^. In the present study, a total of seven rate-limiting enzymes in the flavonoid biosynthesis pathway were significantly altered at the mRNA level in the leaves of *P. alba* × *P. berolinensis* seedlings under the AM fungus treatment, and they were distributed in the three stages mentioned above. Of these, 4CL, CHI, F3H, FLS, and LAR were upregulated, while CHS and ANS were downregulated. These results suggest that AM symbiosis may improve the biosynthesis of flavonols and flavanols and suppress the accumulation of anthocyanidins. Furthermore, TF-encoding gene analysis showed that the transcription factor families involved in flavonoid biosynthesis, including the MYB, bHLH, and NAC families^44–46^, were responsive to mycorrhizal symbiosis, and most TFs of these three families were upregulated in the AMF treatment group. Taken together, the biosynthesis of flavonoids was strongly affected by AMF colonization, which may be the most direct factor underlying the enhancement of resistance to gypsy moth larvae in *P. alba* × *P. berolinensis* seedlings under the AM fungus treatment.

Nontargeted metabolomics technology provides an opportunity to identify the metabolites involved in mycorrhiza-induced resistance in the leaves of *P. alba* × *P. berolinensis* seedlings. Consistent with the results reported by Wang et al.^47^, our results showed that flavonoids were the most abundant class among all the identified metabolites. A discriminant analysis revealed that not all differentially accumulated metabolites with insecticidal properties in mycorrhizal poplar plants were significantly increased. For example, catechin, betaine, and 3-hydroxycinnamic acid, which were previously shown to interfere with insect growth and survival^48–50^, were significantly decreased in the present study. However, several metabolites related to plant resistance to insects, including coumarin, stachydrine, artocarpin, norizalpinin, abietic acid, 6-formylumbelliferone, and vanillic acid, were significantly accumulated in the leaves of mycorrhizal poplar seedlings. These results suggest that AM fungus *G. mosseae* colonization did not improve the chemical defense of *P. alba* × *P. berolinensis* seedlings in a universal fashion. Increases in these insecticidal metabolites may be a direct defense mechanism for the inhibition of *L. dispar* larval growth and survival in the GM treatment group; thus, these compounds might be useful for the development of novel botanical insecticides that can control gypsy moths.

In summary, the AM fungus *G. mosseae*, *P. alba* × *P. berolinensis* seedlings and gypsy moth larvae were successfully employed to analyze the mycorrhiza-induced resistance of a woody plant to a generalist insect herbivore and concluded that AMF inoculation was able to shield *P. alba* × *P. berolinensis* seedlings against *L. dispar* larvae. Further transcriptome and metabolome analyses identified candidate genes and metabolites that are involved in mycorrhiza-induced resistance to insects in the leaves of *P. alba* × *P. berolinensis* seedlings. Special emphasis was placed on the JA signaling pathway and the biosynthesis of flavonoids and differentially accumulated metabolites with insecticidal properties. These findings help to characterize the defense responses in mycorrhizal poplar seedlings.

## Materials and methods

### Plant materials

The AM fungus used in this study was the mycorrhizal inoculum *G. mosseae*, which was purchased from the Gansu Academy of Agricultural Sciences, China. This mycorrhizal inoculum was composed of spores, mycelium, root segments and sand at a concentration of 15 propagules/g mycorrhizal inoculum. *P. alba* × *P. berolinensis* seedlings were annual cuttings purchased from a commercial market in Harbin. The soil substrate for seedlings was composed of turfy soil, vermiculite and sand in a volume ratio of 3:1:1 and was sterilized at 121 °C for 2 h in an autoclave sterilizer before planting the seedlings. In late April 2020, poplar seedlings were planted in pots (16 × 16 × 20 cm) containing a mix of 3 kg sterilized soil substrate with 20 g mycorrhizal inoculum (denoted as GM) or without GM as a noninoculated plant (denoted as CK). A total of 300 poplar seedlings (one plant/pot) were planted (*n* = 150 for each group) and transferred to the plastic greenhouse of Forest Genetic Breeding Nursery in Northeast Forestry University for growth.

### Analysis of root colonization

Root samples of the poplar seedlings were collected 3 months after the plants were inoculated with AMF, washed with sterile water, and stained with 0.008% trypan blue as previously described^13,51^. Mycorrhizal colonization was evaluated under a low-power compound microscope, and the occurrence of spores or arbuscules on the root segments indicated successful colonization. Each group had four replicates, with at least 50 root segments observed for each replicate. The percent mycorrhizal infection was calculated using the following formula and analyzed using an independent sample *t* test (SPSS 19.0 for Windows) at *α* = 0.05. Mycorrhizal infection (%) = 100 × Number of infected root segments/Total number of root segments observed.

### Insect assays

In March 2020, gypsy moth eggs were collected from the Forest Farm of Northeast Forestry University, Harbin, China, and stored at 4 °C before use. In early August, gypsy moth larvae hatched from the eggs, and they were subsequently reared on artificial diets (purchased from Chinese Academy of Forestry, Beijing, China) until they reached 2nd instars. Growth conditions were 25 °C, a 16:8 h (L:D) photoperiod and 60 ± 1% relative humidity in an incubator. The newly molted 2nd instar larvae were divided into two groups and maintained on leaves removed from the nonmycorrhizal and mycorrhizal poplar seedlings. Leaves were refreshed every 2 days until the larvae died or pupated. Each group contained 80 larvae, and these larvae were reared in 10 plastic boxes (18 × 8 × 6 cm). The numbers of dead and live larvae were recorded, and survival curves were generated using SPSS 19.0 software for Windows and analyzed by the log-rank test for significance. The body weight, body length, head capsule width and developmental duration of 3rd-5th instar larvae in the CK and GM groups (*n* = 24 in each group) were also recorded and analyzed using an independent sample *t* test at *α* = 0.05.

### Transcriptome analysis

In mid-August 2020, the first five leaves from the top of each of three healthy seedlings were removed and then combined for a replicate. A total of three replicates were generated for each group (GM or CK). All poplar seedlings used for the transcriptome analysis had not been previously harvested for the rearing of gypsy moth larvae or for performing other experiments. The experimental process for the transcriptome analysis was conducted by LC-Bio Technology Co., Ltd. (Hangzhou city, China) according to the standard provided by the Illumina Company (San Diego, CA, USA), including library preparation and sequencing experiments. Total RNA from each poplar leaf sample was isolated using TRIzol reagent (Invitrogen, CA, USA) following the manufacturer’s instructions. The total RNA quantity and purity were checked using a Bioanalyzer 2100 and RNA 1000 Nano LabChip Kit (Agilent, CA, USA) with RIN number >7.0. Poly(A) RNA was purified from total RNA (5 µg) over two rounds using poly-T oligo-attached magnetic beads. After purification, the mRNA was fragmented into small pieces using divalent cations under an elevated temperature. Then, the cleaved RNA fragments were reverse-transcribed to create the final cDNA library using the mRNA Seq sample preparation kit (Illumina) in accordance with the manufacturer’s instructions, and the average insert size for the paired-end libraries was 300 bp (±50 bp). Paired-end sequencing was conducted on an Illumina HiSeq 4000 platform in accordance with the recommended protocol.

To acquire high-quality reads, in-house developed Cutadapt and Perl scripts were used to remove adapter sequences, low-quality reads and reads containing poly-N^52^. The sequence quality of the clean data was verified using FastQC, including the Q20, Q30, and GC content. All downstream analyses were based on clean data with high quality. De novo assembly of the transcriptome was accomplished with Trinity 2.4.0 software. All assembled unigenes were annotated using the following databases: SwissProt, GO, Nr, KEGG, and eggNOG. The expression level of each gene was analyzed using RSEM and normalized to the fragments per kilobase of transcript sequence per million base pairs sequenced (FPKM) value. Genes with an absolute value of |log2FC|> 1 and with statistical significance (*P* value < 0.05) using the R package edgeR were defined as differentially expressed genes (DEGs). GO and KEGG enrichment analyses of the DEGs were carried out with padj < 0.05 to identify the significantly enriched pathways.

### Real-time PCR

To verify the transcriptomic data results, 12 DEGs were randomly selected and their mRNA abundances were analyzed via real-time PCR, as previously described^53^. In brief, cDNA was synthesized with RNA used in transcriptome analysis by the PrimeScript™ RT Reagent Kit (Takara, China), and RT-PCR was performed using the StepOnePlus™ Real-Time PCR System (Thermo) and the fluorescence dye SYBR Premix Ex TaqTM (TaKaRa, China). Each RT-PCR assay (10 µL) contained 5 µL of SYBR Premix Ex TaqTM, 0.4 µL of each primer (0.5 mmol/L), 1 µL of template cDNA, 0.2 µL of ROX Reference Dye and 3 µL of ddH_2_O. The amplification program included 95 °C for 30 s, followed by 95 °C for 5 s and 60 °C for 30 s (40 cycles). There were three biological replicates for each gene. The EF1β gene was used as a reference gene for normalization^54^, and the gene-specific primers for RT-PCR are shown in Supplementary Table S5. The relative gene expression was calculated according to the 2^−△△Ct^ method.

### Metabolome analysis

In mid-August 2020, the first five leaves from the top of each of three healthy seedlings were removed and then combined for a replicate. A total of six replicates were established for each group (GM or CK). All poplar seedlings used for metabolome analysis had not been previously harvested for rearing gypsy moth larvae or performing other experiments. The metabolome analysis was performed by LC-Bio Technology Co., Ltd. (Hangzhou city, China) using the ultra-performance liquid chromatography quadrupole time-of-flight mass spectrometry (UPLC-QTOF MS) method. In brief, poplar leaf samples were frozen in liquid nitrogen and then ground. Metabolites from the ground leaf samples were extracted with 50% methanol. One microliter of the metabolite extract was injected into an Acquity UPLC system coupled to a TripleTOF 5600 plus high‐resolution tandem mass spectrometer (SCIEX, UK) and separated with an ACQUITY UPLC T3 column (100 mm×2.1 mm, 1.8 µm, Waters, UK). LC-MS raw data files were converted into mzXML format using Proteowizard MSConver software and then processed by the XCMS, CAMERA, and metaX toolboxes implemented with R software^55^. The online KEGG and HMDB databases were used to annotate the metabolites by matching the exact molecular mass data (*m*/*z*) of samples with those from the database. Student’s *t* tests were conducted to detect differences in metabolite concentrations between the control and treatment groups. The *P* value was adjusted for multiple tests using an FDR (Benjamini–Hochberg Method). Multivariate data analyses, including PCA and PLS-DA, were conducted using metaX to discriminate the different variables between groups. The VIP value was calculated. Differentially expressed metabolites (DEMs) among the CK and GM groups were defined at VIP > 1 and *P* < 0.05.

### Plant growth determination

In late August 2020, 12 unplucked poplar seedling plants in the CK or GM group were harvested and divided into roots, stems, and leaves by group. Their growth parameters, including the root length and plant height, as well as biomass parameters, including the fresh weights and dry weights of below- and aboveground parts, were recorded. Data were analyzed using an independent sample *t* test at *α* = 0.05.

## Supplementary information

Supplementary files

## Data Availability

The RNA-sequencing data have been deposited in the NCBI Sequence Read Archive (SRA, http://www.ncbi.nlm.nih.gov/sra) database, with the accession number PRJNA753556. The other referenced data are included in the article or Supplementary Materials.
