# Supplementary material for: Defense responses of arbuscular mycorrhizal fungus-colonized poplar seedlings against gypsy moth larvae: a multiomics study
Source: Hortic Res. 2021 Dec 1;8:245. doi: 10.1038/s41438-021-00671-3 (PMC8632881; doi:10.1038/s41438-021-00671-3)
Supplement: Supplementary file 1 — Supplementary files [file 41438_2021_671_MOESM1_ESM.doc]

**Supplementary files**

**Table legends**

Table S1 Growth status of mycorrhizal and nonmycorrhizal poplar plants

Table S2 Number of live individuals at different times for gypsy moth larvae fed on the leaves of mycorrhizal or nonmycorrhizal poplar plants.

Table S3 Summary of transcriptome data from leaves of *Populus alba* ×*P. berolinensis* seedlings.

Table S4 The top 5 GO terms with the most significant enrichment in cellular component, biological process or molecular function, respectively.

Table S5 Sequences of RT-PCR primers in the present study

**Figure legends**

Fig.S1 Arbuscular mycorrhizal (AM) fungal structures (A) and colonization rate of AM fungus (B) in root segments of mycorrhizal poplar seedlings.

Fig.S2 The Venn diagram of genes annotated in Nr, Swissprot, Pfam, eggNOG, GO and KEGG databases.

Fig.S3 Differentially expressed genes (DEGs) in the leaves of mycorrhizal poplar seedlings. Number DEGs in mycorrhizal poplar seedlings compared with the un-treated control (A). Volcano plots showing the DEGs among GM and CK groups (B). GM, *Glomus mossae*-treated group. CK, un-treated group.

Fig.S4 Comparison of the relative transcriptional levels of RNA-seq and qPCR results. Bars represent means ± standard deviations (n = 3).

Fig.S5 Bubble chart of the top 30 enriched pathways in KEGG analysis of differentially expressed genes.

Fig.S6 The top 15 kinds of metabolites at the class level in leaves of *Populus alba* ×*P. berolinensis* seedlings.

Fig.S7 Derived PCA score plot (A), derived OPLS-DA score plots (B), and the corresponding validation plots (C) of OPLS-DA from metabolite profiles in leaves of *Populus alba* ×*P. berolinensis* seedlings.

Fig.S8 Pathway analysis of the differentially expressed metabolites among *Glomus mossae*-treated group (GM) and un-treated group (CK).

Table S1 Growth status of mycorrhizal and nonmycorrhizal poplar plants

| Treatment | Growth index (cm plant^-1^) | | Aboveground weight (g plant^-1^) | | Underground weight (g plant^-1^) | |
| --- | --- | --- | --- | --- | --- | --- |
|  | Plant height | Root length | Fresh | Dry | Fresh | Dry |
| CK | 90.25±4.05a | 40.47±0.12a | 51.89±2.13a | 26.15±0.92a | 5.22±0.10a | 2.43±0.12a |
| GM | 87.24±3.64a | 46.20±0.54b | 48.71±1.10a | 24.82±0.88a | 6.01±0.11b | 3.60±0.18b |

Values presented in the table are means±standard deviations (n = 9). Different lowercases within the same column mean a significant difference among CK and GM group at *α* level of 0.05. GM, Glomus mossae-treated group. CK, un-treated group.

Table S2 Number of live individuals at different times for gypsy moth larvae fed on the leaves of mycorrhizal or nonmycorrhizal poplar plants.

| Treatment | Days after treatment | | | | | | | | | | | | | Survival rate |
| --- | --- | --- | --- | --- | --- | --- | --- | --- | --- | --- | --- | --- | --- | --- |
|  | 3 | 4 | 5 | 6 | 7 | 8 | 9 | 10 | 11 | 12 | 13 | 14 | 15 |  |
| CK | 80 | 80 | 80 | 79 | 79 | 79 | 79 | 78 | 78 | 78 | 78 | 78 | 78 | 97.5% |
| GM | 80 | 77 | 75 | 72 | 69 | 65 | 62 | 60 | 59 | 59 | 59 | 59 | 59 | 73.75% |

Table S3 Summary of transcriptome data from leaves of *Populus alba* ×*P. berolinensis* seedlings.

| Transcriptome date |  |  |
| --- | --- | --- |
| **Output results** | Control group | Treatment group |
| Clean reads | 41900971 | 39119217 |
| GC percentage | 45.76 % | 45.72 % |
| Q20 of clean reads | 98.31 % | 98.26% |
| Q30 of clean reads | 94.51 % | 94.46% |
| **Assembly results of transcripts** |  | |
| Transcripts number | 127960 | |
| Total Nucleotides (bp) | 135316228 | |
| N50 length (bp) | 1566 | |
| Mean length (bp) | 1057 | |
| Largest length (bp) | 16491 | |
| **Assembly results of genes** |  | |
| Gene number | 46397 | |
| Total Nucleotides (bp) | 44387562 | |
| N50 length (bp) | 1597 | |
| Mean length (bp) | 957 | |
| Largest length (bp) | 16491 | |

Table S4 The top 5 GO terms with the most significant enrichment in cellular component, biological process or molecular function, respectively.

| GO_ID | GO Term | GO Function | GO Level | p-value |
| --- | --- | --- | --- | --- |
| GO:0009579 | thylakoid | Cellular component | 5 | 0 |
| GO:0009570 | chloroplast stroma | Cellular component | 8 | 1.11E-16 |
| GO:0009534 | chloroplast thylakoid | Cellular component | 7 | 1.11E-16 |
| GO:0009535 | chloroplast thylakoid membrane | Cellular component | 8 | 2.22E-16 |
| GO:0009941 | chloroplast envelope | Cellular component | 7 | 2.22E-16 |
| GO:0015979 | photosynthesis | Biological process | 5 | 2.22E-16 |
| GO:0055114 | oxidation-reduction process | Biological process | 4 | 4.59E-12 |
| GO:0015995 | chlorophyll biosynthetic process | Biological process | 9 | 2.89E-09 |
| GO:0006857 | oligopeptide transport | Biological process | 9 | 9.37E-09 |
| GO:0005975 | carbohydrate metabolic process | Biological process | 5 | 2.14E-08 |
| GO:0004553 | hydrolase activity, hydrolyzing O-glycosyl compounds | Molecular function | 6 | 2.43E-07 |
| GO:0016168 | chlorophyll binding | Molecular function | 6 | 4.32 E-06 |
| GO:0016491 | oxidoreductase activity | Molecular function | 4 | 2.45 E-05 |
| GO:0005355 | glucose transmembrane transporter activity | Molecular function | 9 | 7.54 E-05 |
| GO:0004497 | monooxygenase activity | Molecular function | 5 | 9.01 E-05 |

Table S5 Sequences of RT-PCR primers in the present study

| Genes | TM | Product size | Primer efficiency | Primer sequence (5' - 3') |
| --- | --- | --- | --- | --- |
| DN10023_c0_g1 | 60 ℃ | 101 | 1.04 | Forward:AACATGGGCAGGGACAAGTG |
|  |  |  |  | Reverse:AGACGCCAACGACTGATCATA |
| DN10025_c0_g1 | 60 ℃ | 101 | 1.00 | Forward: TCAAGTCGAGGTCACAGCTT |
|  |  |  |  | Reverse: ATGAAAGCGTGGTAATGGCC |
| DN10125_c0_g1 | 60 ℃ | 90 | 0.96 | Forward: ACAGCAAGCCTTGTGTTAGC |
|  |  |  |  | Reverse: GCTTCTGAATGGACACTGCC |
| DN10137_c0_g1 | 60 ℃ | 139 | 0.98 | Forward: TGGGTCGTATCGCTAGGTTC |
|  |  |  |  | Reverse: TTGTCTCTTGCTGCGTTTCC |
| DN10140_c1_g2 | 60 ℃ | 106 | 0.95 | Forward: ATGGGAGCAGAAGTTGTGGA |
|  |  |  |  | Reverse: TCCAATGGAGAGCTGGGTTT |
| DN10167_c0_g1 | 60 ℃ | 145 | 1.01 | Forward: GGGTTTCATGTGGGGAGTCT |
|  |  |  |  | Reverse: CTTCGGACATGGCATTCACC |
| DN10212_c0_g1 | 60 ℃ | 135 | 0.97 | Forward: TCCTCCACCAATCTCTTGCC |
|  |  |  |  | Reverse: CGGTTAGCACGCAAAATGTC |
| DN10276_c0_g1 | 60 ℃ | 144 | 0.94 | Forward: CGGTCACCACAAATCCATCC |
|  |  |  |  | Reverse: CATTGCCAATCCCACCTAGC |
| DN10294_c0_g1 | 60 ℃ | 167 | 1.06 | Forward: CTGCTTCCACCTCCTCATCA |
|  |  |  |  | Reverse: CATTGTTGGCCGATAGTGGG |
| DN10295_c0_g2 | 60 ℃ | 133 | 0.97 | Forward: TCCCAGTACCCGAATTCCTT |
|  |  |  |  | Reverse: CTACCATACCCGAGTCCACC |
| DN10352_c0_g1 | 60 ℃ | 178 | 0.98 | Forward: ATGTGGATGTGGTGGAGGAG |
|  |  |  |  | Reverse: TCAGCTGGTTTTCTCCGTCT |
| DN10418_c0_g1 | 60 ℃ | 94 | 0.97 | Forward: CTTCTTGTCTGTGCTCGCTC |
|  |  |  |  | Reverse: AATCCCATCCATCCCAGCAA |
| EF1βR | 60 ℃ | 145 | 0.99 | Forward: AAGAGGACAAGAAGGCAGCA |
|  |  |  |  | Reverse: CTAACCGCCTTCTCCAACAC |

Fig.S1 Arbuscular mycorrhizal (AM) fungal structures (A) and colonization rate of AM fungus (B) in root segments of mycorrhizal poplar seedlings.


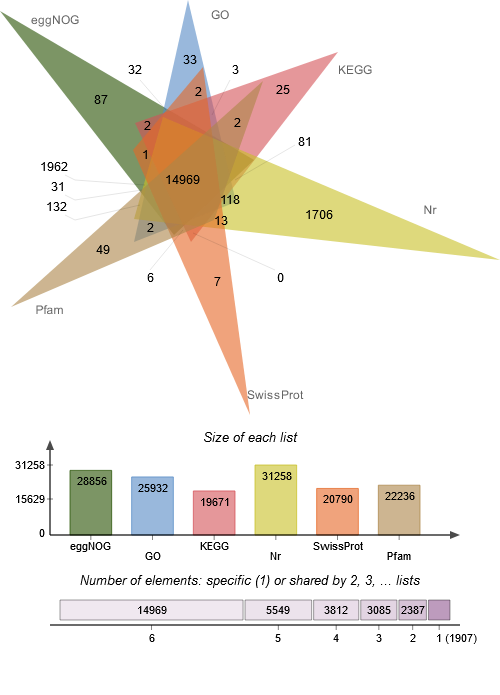


Fig.S2 The Venn diagram of genes annotated in Nr, Swissprot, Pfam, eggNOG, GO and KEGG databases.


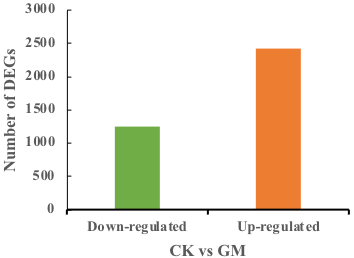

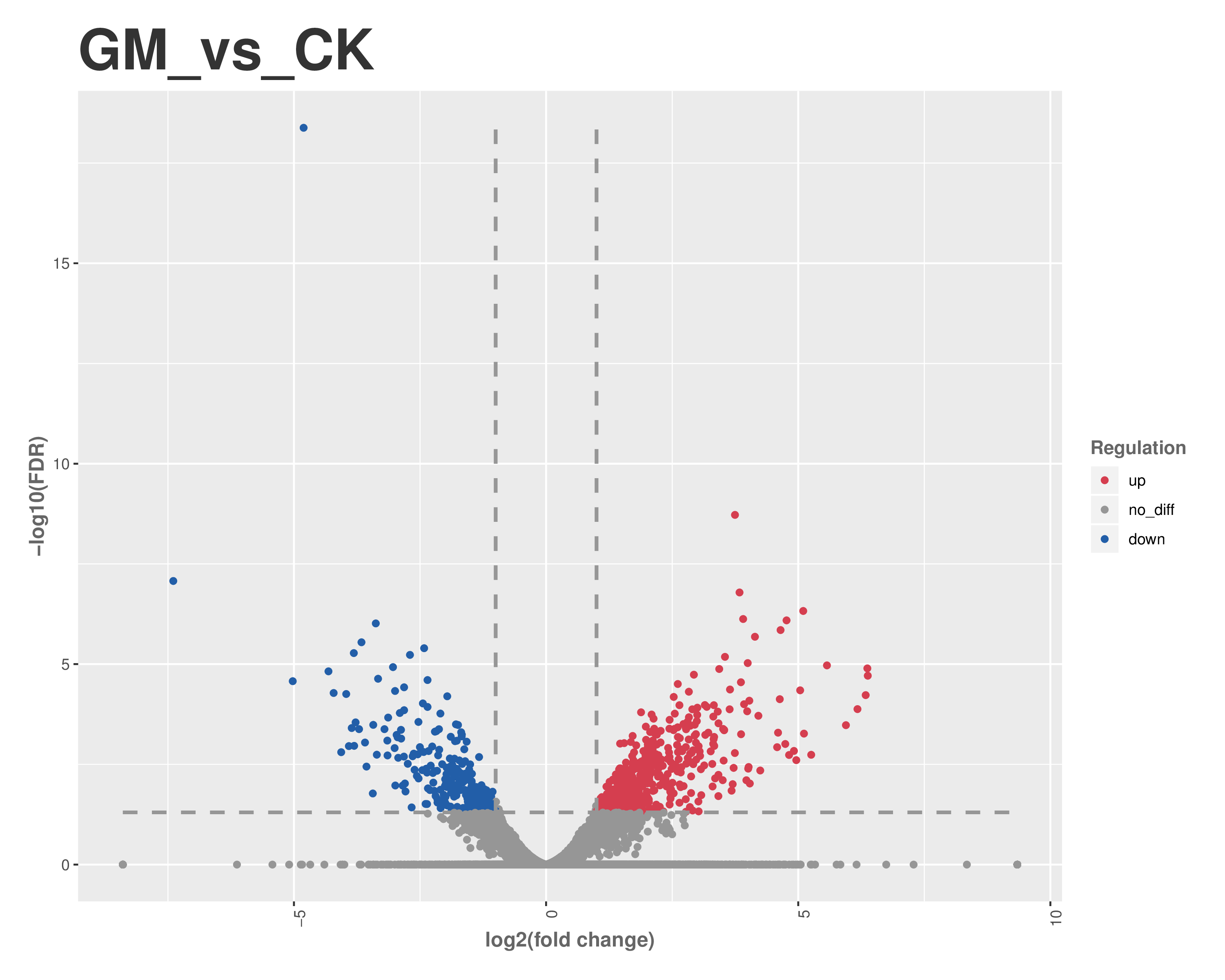


A

B

Fig.S3 Differentially expressed genes (DEGs) in the leaves of mycorrhizal poplar seedlings. Number DEGs in mycorrhizal poplar seedlings compared with the un-treated control (A). Volcano plots showing the DEGs among GM and CK groups (B). GM, *Glomus mossae*-treated group. CK, un-treated group.

Fig.S4 Comparison of the relative transcriptional levels of RNA-seq and qPCR results. Bars represent means + standard deviations (n = 3).


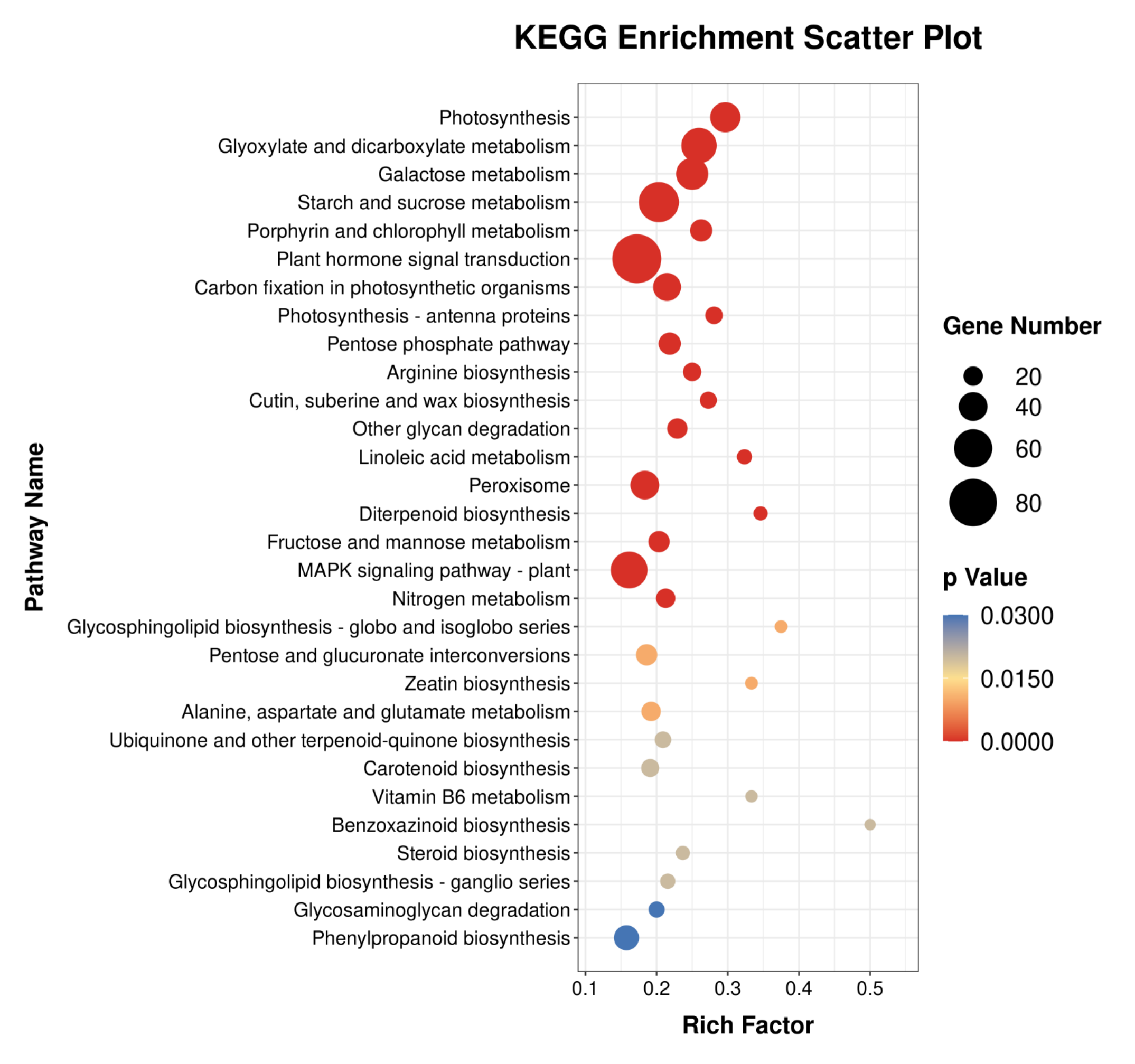


Fig.S5 Bubble chart of the top 30 enriched pathways in KEGG analysis of differentially expressed genes.


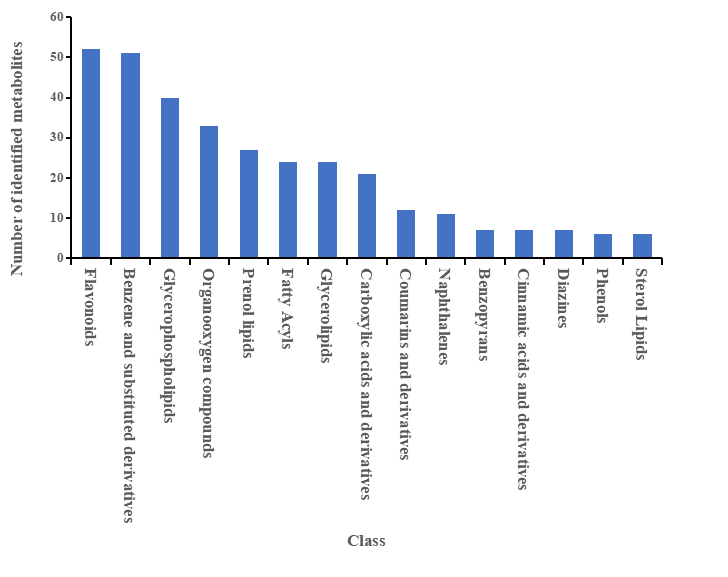


Fig.S6 The top 15 kinds of metabolites at the class level in leaves of *Populus alba* ×*P. berolinensis* seedlings.


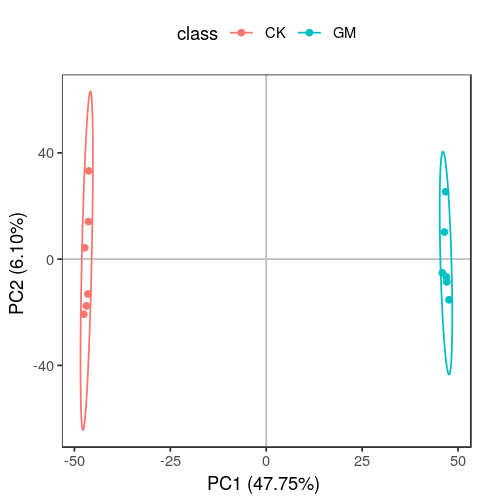

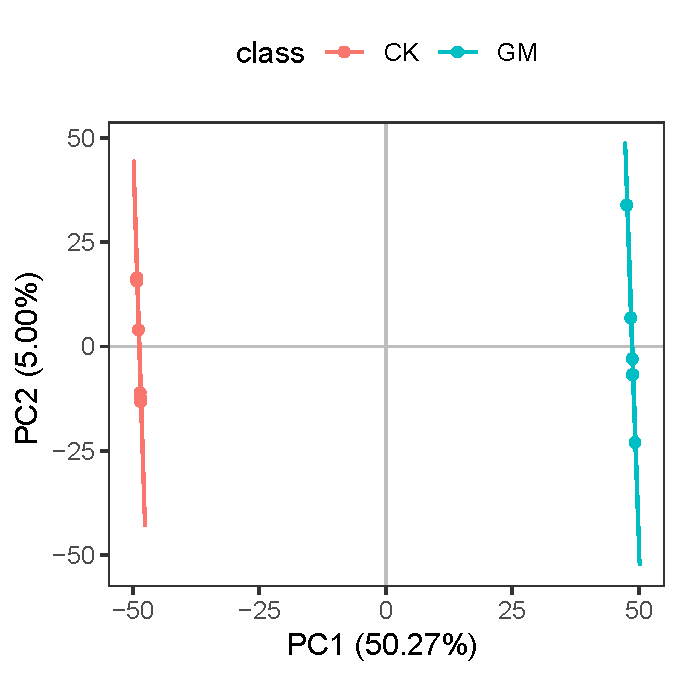

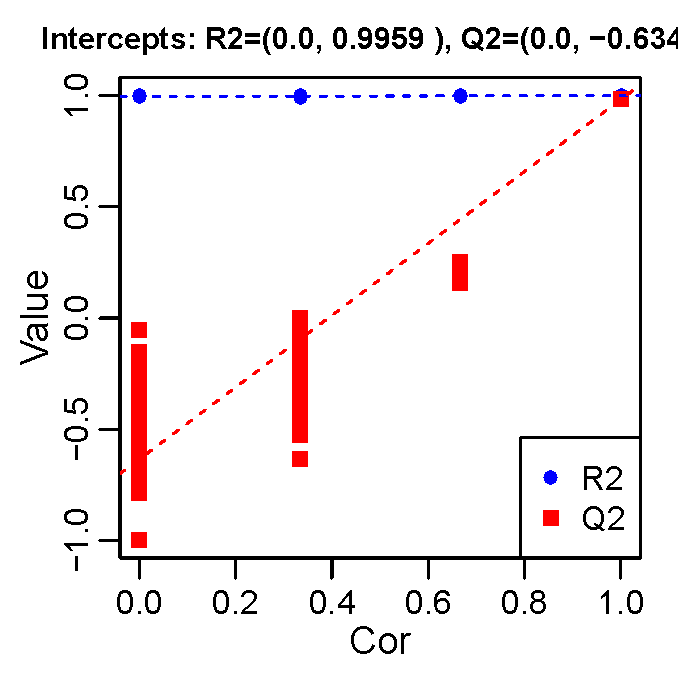


A

B

C

Fig.S7 Derived PCA score plot (A), derived OPLS-DA score plots (B), and the corresponding validation plots (C) of OPLS-DA from metabolite profiles in leaves of *Populus alba* ×*P. berolinensis* seedlings.


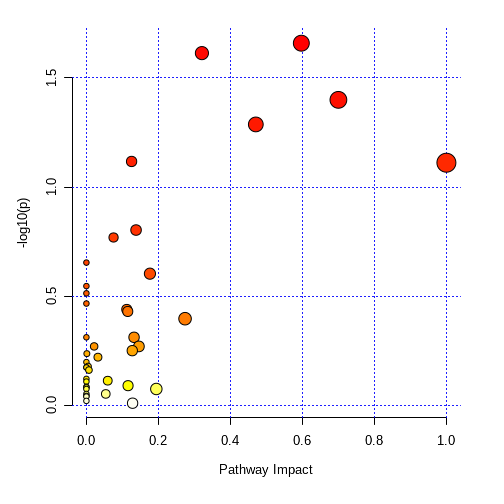


Isoquinoline alkaloid biosynthesis

Flavone and flavonol biosynthesis

Glycerolipid metabolism

Phenylalanine metabolism

Glycerophospholipid metabolism

Fig.S8 Pathway analysis of the differentially expressed metabolites among *Glomus mossae*-treated group (GM) and un-treated group (CK).
